# The ethical considerations of primordial pandemic prevention from a one health perspective

**DOI:** 10.1186/s13010-025-00166-2

**Published:** 2025-03-12

**Authors:** Rebecca Shalansky, Ross Upshur

**Affiliations:** Dalla Lana School of Public Health, The University of Toronto, 155 College St, Toronto, ON M5T 3M7 Canada

**Keywords:** One Health, Public health, Global health, Ethics, Pandemic, Zoonotic disease, Primordial prevention, Primary prevention

## Abstract

**Background:**

The coronavirus disease 2019 (COVID-19) pandemic has left a devastating global toll. As such, there is a strong impetus to prevent future global pandemics. Ethical considerations are an integral element of pandemic preparedness and response plans and should be incorporated into any pandemic prevention plan to explicitly examine the incorporated values from various stakeholders. Our study aims to determine the ethical considerations of primordial pandemic prevention from a One Health perspective.

**Methods:**

This was a prospective Delphi consensus seeking-study. We aimed to recruit a purposive, globally representative sample of experts in the fields of public health ethics, One Health ethics, pandemic ethics and pandemic prevention. Two rounds were completed between November 2021, and January 2022. The first round consisted of open-ended questions to establish ethical considerations for primordial pandemic prevention. Thematic analysis was used to uncover themes. The second-round presented the ethical consideration results of the first round, and asked participants to rate the importance of each of them.

**Results:**

The first-round had 27 participants, and the second-round had 25 participants. Both rounds had global representation from all intended fields of expertise. There were five ethical considerations for which consensus was achieved: Promoting equity, global collective effort, distributive justice, evidence-based efficiency and the interconnectedness of humans, animals and the environment.

**Conclusions:**

Our study identified five ethical considerations for primordial pandemic prevention from a globally representative sample. The findings will contribute to current and future pandemic prevention policy, and expand ethics research in the fields of One Health, pandemic prevention and zoonotic disease control.

**Supplementary Information:**

The online version contains supplementary material available at 10.1186/s13010-025-00166-2.

## Introduction

### Background

The COVID-19 pandemic left a devastating global toll, including mass morbidity, mortality and economic impact. At the time of writing, there have been over 7 million reported COVID-19 deaths according to the World Health Organization (WHO) [1, 2]. As such, there is a strong global impetus, including by the WHO, the Organization for Economic Co-operation and Development (OECD) and other global leaders, to prevent, or at least reduce the impact of, future pandemics [1–4]. “Pandemic” is defined as a rise in the incidence of illness clearly in excess of normal expectation occurring over much of the world [5]. Criticism of current pandemic planning is that it is reactive, rather than proactive [1]. The Intergovernmental Science-Policy Platform on Biodiversity and Ecosystem Services (IPBES), report on biodiversity and pandemics explains that existing pandemic prevention “is based on containment and control after a disease has emerged…rather than on reducing the drivers of pandemic risk to prevent them before they emerge” [1]. To effectively prevent global pandemics, primordial prevention should be implemented, which is defined by the Association of Faculties of Medicine of Canada (AFMC) as preventing an outcome by influencing “population health determinants and inhibit[ing] the establishment of factors (environmental, economic, social, behavioural) known to increase the future risk of disease” [6].

The WHO has commenced drafting an intergovernmental agreement on pandemic prevention, preparedness and response “because the COVID-19 pandemic has shown that the status quo is not good enough to protect our communities, our societies, and our economies” [7, 8]. We argue that such efforts require the identification of the ethical considerations to make explicit the tacit values that go into existing and emerging pandemic prevention frameworks, such as the WHO’s; Otherwise, the various stakeholders may speak at cross purposes due to unarticulated conflicting ethical values.

Approximately 75% of emerging infectious disease outbreaks are of zoonotic origin, including those that have led to major pandemics such as HIV, Ebola, Zika, avian & swine influenza, SARS-CoV, MERS and most likely SARS-CoV-2 [9]. Globally, there are many human-driven practices known to increase the risk of animal to human disease transmission which could initiate a pandemic of zoonotic origin. Examples include the consumption wild meat, mass deforestation leading to loss of biodiversity, factory farming, and live markets [1].

Ethical considerations are especially important for the topic of zoonotic originated pandemic prevention, as many practices at high risk for zoonotic transmission are currently essential to the populations that rely on them for their cultural, economic and nutritional needs [10, 11]. One must also consider that zoonoses commonly emerge in tropical low and middle income countries (LMIC), where populations are already vulnerable, and may face stigma [1, 12].

As zoonotic diseases involve both human and animal health, it has been argued that a pandemic prevention framework should incorporate a One Health lens [12, 13]. One Health is defined by the WHO as “an integrated, unifying approach to balance and optimize the health of people, animals and the environment [14]. Though One Health traditionally separates “people” and “animals,” it is important to note that homo sapiens are also animals. Both the United Nations Environment Programme (UNEP) and International Livestock Research Institute (ILRI) and the IPBES reports on pandemic prevention emphasize the importance of a One Health approach [1, 12]. As per the UNEP ILRI report: “Adopting a One Health approach…will help governments, businesses and civil society achieve enduring health for people, animals and environments alike” [12].

Because of the complex relationship between pandemic prevention and human behaviour, the inequitable distribution of burden on LMIC, and the interaction between animals, humans and their environments, a deeper understanding of the ethical issues informing pandemic prevention could harmonize public health, global health and One Health ethics, as well as have its own unique values. This unique perspective on pandemic prevention ethics has been called for in the literature, exemplified by Diller and Williamson’s article quite clearly titled “Supporting One Health for Pandemic Prevention: The Need for Ethical Innovation” [15]. Without an integrated ethical framework, we are at risk of limiting ourselves to only currently available frameworks, with anthropocentric foundations, and we will miss the “developments required to create and maintain relationships able to sustain environmental and human health” [15]. The research question of this study is: What are the ethical considerations of primordial pandemic prevention from a One Health perspective?

### Existing literature

A background review of the literature by the authors found minimal survey or interview-based research on ethical considerations of pandemic prevention. Our review found a small number of research studies soliciting input on the ethics of emerging zoonotic disease control. These findings are relevant in the study of pandemic prevention ethics given the frequent zoonotic origin of global pandemics [9]. Lysaght et al. conducted a modified Delphi to determine the ethical priorities for the management of zoonotic emerging infectious disease in Singapore [16]. The principle of justice, both related to the human-animal health tension as well as the global and regional responsibilities, was highlighted [16]. From semi-structured interviews of specialists in zoonotic disease control in the Netherlands, van Herten et al. determined that the human-animal health tension, as well as honesty and transparency, were important considerations in zoonotic disease control [17]. The original Beauchamp and Childress bioethical principles (beneficence, non-maleficence, justice and autonomy) were also highlighted in the van Herten study [17, 18].

Beyond primary literature, there are broader discussions of ethical considerations of pandemic prevention, typically in commentaries, organizational statements and policy. For example, equity, especially between economically disparate countries, has been argued to be a key factor to incorporate into the WHO’s pandemic agreement, with a recent publication stating “promises of equity and cooperation must remain during negotiation, but they must also be implemented by all, even in the face of powerful lobbying from vested commercial and political interests” [19]. Though not specifically stated, the principle of distributive justice is also evident from this commentary. Justice, specifically distributive and ecojustice, are also emphasized by Diller and Williamson who state regarding One Health that “justice needs to be equipped to work with a far greater array of diversity than it is currently accustomed…” emphasizing a move away from anthropocentric justice [15].

Additionally, The first draft of the WHO’s pandemic agreement itself incorporates One Health, though mostly from an anthropocentric lens [8]. This brings up one of the central ethical questions considered in One Health literature - the innate value of animals and the environment, beyond their benefit to humanity [13, 15, 17]. As van Herten, Bovenkerk, Verweij articulate “To justify zoonotic disease control measures like the culling of healthy animals, professional health workers and policy makers should make their underlying moral presuppositions about the moral status of animals more explicit” [13].

## Methods

### Study design

This was a prospective Delphi consensus seeking-study. The Delphi method was developed in the 1950’s by the RAND corporation as a tool for forecasting and decision-making [20–22]. The process consists of gathering a group of subject experts, traditionally blinded to each other, and sequentially providing question rounds with unidentified information from the past round’s results in order obtain agreement [20]. The rounds are continued until consensus is achieved, often involving two to three rounds [23]. It is a flexible, low-barrier study design, and it allows for the collection of both quantitative and qualitative data [24]. The Delphi method was ideal for this study due to the novelty of its subject, and the international demographic of experts in the field.

### Participants

We aimed to recruit a purposive (non-random), globally representative sample of professional experts in the fields of public health ethics, One Health ethics, pandemic ethics and pandemic prevention. The ideal number of participants, and the ideal criteria for selecting participants in a Delphi study is not clearly defined in the literature [23]. Based on desiring a wide representation of respondents in a narrow field, we aimed for 15–20 participants.

Participants were identified first by authorship of relevant articles found in a literature search by the primary author (unpublished research), second by membership in the WHO Collaborating Centres for Bioethics Network, the WHO Ethics and COVID-19 Working Group and the WHO Access to COVID-19 Tools Accelerator Ethics and Governance Working Group, and third by professional connections of the authors, who have expertise in public health, pandemic prevention and biomedical ethics. Additionally, in order to increase study participation, participants were invited to forward the study invitation to relevant colleagues, as per the exponential, non-discriminative snowball technique [25]. Participants were aware of the fields of expertise the study was seeking input from. There was no compensation for participation in any part of our study, including for referring the study to others. The use of the snowball technique removed the full anonymity of participants to each other.

Participants were invited to the first-round in November 2021, and the second-round in January 2022. For each round, participants had two weeks to respond. A reminder email was sent one week after the invitation. Each invitation contained a preamble describing the nature of the study, including the AFMC definition of primordial prevention [6]. The same group was invited for both rounds, regardless of participation in the first. This meant the second-round was not necessarily completed by the same cohort as the first. Responses were anonymous to the researchers, and were unlinked to individual participants. Written informed consent was obtained for both rounds, and participants were made aware that they could withdraw their consent to participate at any time.

### Question rounds

To collect and manage responses, the REDCap electronic data capture tool hosted at the University of Toronto, version 12.0.18, was used [26, 27]. Both rounds were conducted in English. The first round contained three open-ended questions:

1. What are 3–5 general ethical considerations (if any) that should be included in primordial pandemic prevention policy?
2. Which are 3–5 global health ethical considerations (if any) that should be included in primordial pandemic prevention policy?
3. What are 3–5 animal and/or environmental ethical considerations (if any) that should be included in primordial pandemic prevention policy?

As per the Delphi method, once the results of the first-round were analyzed, the second-round was developed based on its findings [23]. Controlled feedback was provided to the participants in the second-round by presenting the ethical consideration results of the first-round with a description (See supplemental material for specific controlled feedback provided). Participants were not provided with the total number of times each ethical consideration (theme) was mentioned by the first-round participants. It was instructed that the ethical considerations were not listed in any meaningful order. Participants were instructed to rate the importance of each ethical considerations on a 7-point Likert scale. Multiple ethical considerations could receive the same numerical value if they were considered of equal importance level by the participants. There was also space in the second-round for open-ended commentary, including whether there were any ethical considerations missing from this list. As consensus (definition described below) was achieved after two rounds, a third round was not completed. The questions can be accessed in full as supplemental material.

### Statistical analysis

The responses to the first-round were entirely open-ended. For analysis, the six-phase thematic analysis process developed by Braun and Clarke was used, supplemented by the Association for Medical Education in Europe thematic analysis of qualitative data guide [28, 29]. The primary author first familiarized herself with the open-ended data, then generated initial codes. Once codes were generated, themes of broader significance were determined, and then reviewed. Themes were then named and defined [28, 29]. Finally, the total number of times each theme was mentioned was tallied. Totals for each theme may have been higher than the total number of participants, as there were three open-ended questions per participant, which could have contained the same themes. All thematic analysis was performed in Microsoft Word for Mac, version 15.33.

In the second-round each consideration was ranked from most to least important based on the added total score of all participants’ ratings. A rating of 7 on Likert scale equated to score of 7, 6 a score of 6, etc. As a fictional example, if there was a total of three study participants, and they each ranked ethical consideration X as 5, 4 and 7 respectively, the total score for that consideration would be 16 (5 + 4 + 7).

Based on Soriano et al.’s Delphi methodology, consensus that an ethical consideration was important was determined to be achieved if the percentage of respondents who rated the consideration between 5 and 7 was greater than 70% [30]. Disagreement was determined if 35% or more of the responses for an ethical consideration fell within 1–3 and 35% or more of the responses fell within 5–7 (both extremes of possible options). Partial agreement was concluded for all other combinations [30]. These definitions were determined in advance of the first survey round. All quantitative analysis was performed in Microsoft Excel for Mac version 15.33.

There were insufficient responses to the open-ended questions of the second-round to conduct a thematic analysis.

### Ethics

Ethics approval was obtained by the University of Toronto Health Science Human Research Ethics Board in August 2021, protocol number 25392.

## Results

### Participant information

On November 3, 2021, 135 emails were successfully sent to unique email addresses inviting individuals to participate in the first-round. On January 11, 2022, the same email addresses were sent invitations to the second-round. Since participants were encouraged to forward the study invitations to relevant colleagues, the total number of invitation recipients is unknown.

The first-round had 27 participants, and the second-round had 25 participants. Both rounds had representation from all intended fields of expertise, with public heath ethics being the predominant field for both rounds at 48% and 72% respectively. The first-round had representation from all continents other than South America and Antarctica. The second-round had all continents but Antarctica represented. In both rounds, the majority of the participants’ location of practice was listed as Asia at 44% and 40% respectively. At least 75% of participants in both rounds had ten or more years of experience in their fields of expertise. The complete demographic information of participants is displayed in Table 1.

**Table 1:** Participant demographics

	Round One (*N* = 27)	Round Two (*N* = 25)
**Field of Expertise** (select all that apply)		
One Health Ethics	11 (41%)	10 (40%)
Public Health Ethics	13 (48%)	18 (72%)
Global Health Ethics	11 (41%)	13 (52%)
Pandemic Prevention	7 (26%)	6 (24%)
Other	8 (30%)	7 (28%)
**Location of Practice** (select all that apply)		
Africa	6 (22%)	3 (12%)
Antarctica	0	0
Asia	12 (44%)	10 (40%)
Europe	9 (33%)	3 (12%)
North America	4 (15%)	8 (32%)
Oceana	6 (22%)	3 (12%)
South America	0	2 (8%)
**Years of Experience**		
< 5	1 (4%)	0
5–10	5 (19%)	5 (20%)
10–20	13 (48%)	9 (36%)
> 20	8 (30%)	11 (44%)
Total	27 (100%)	25 (100%)

### First-round

Eight themes were identified from the review of responses to the first-round. The below list is presented in order of the frequency a theme was noted by participants. Where there is a tie, there is no meaningful order to which is listed first.

1. Interconnectedness of humans, animals and the environment:

Human, animal and environmental health overlap. There was a range of consideration about whether the overlap is important to consider for the ultimate outcome of human health, or whether to also consider the impact on animal and environmental wellbeing.

*“Adopt an ecosystems perspective on prevention (avoiding separating human health from ecosystem health)”*

*“Invasive procedures on ecosystems and their wildlife need to be avoided at all cost, all the more given that ecosystem degradation and wildlife diseases ultimately contribute to human health risks”*

*“De-forestation and human encroachment on habitat is a key driver of both climate change and emerging infectious diseases with pandemic potential. If this is not addressed, any prevention policy is bound to fail”*

2. Distributive Justice.

The benefits and burdens of pandemic prevention must be distributed in a manner that is fair based on the ethical considerations in this list. This could include between nations, groups of people, generations, species etc. The response of “justice,” twice with no further elaboration, was provided eight times.

*“…resources should be allocated fairly among people*,* with an emphasis on vulnerable populations*”

*“Fair distribution of benefits and burdens imposed on the society by measures of primordial prevention;*”

3. Communities as stakeholders:

Community members must be included as stakeholders in decision making. Methods of doing so include active engagement with the community, public education, maintaining public trust and being publicly transparent in decision-making. Incorporating communities was also tied to the fourth theme of promoting equity, as community includes involving those who are most marginalized.

*“Priority setting by impacted stakeholders, not just experts”*

*“Community consent fostered by sustained, accurate communication”*

*“Ensuring public involvement and transparency in the process of planning primordial prevention…”*

*“Community or public engagement including the marginalized”*

4. Promoting equity.

In the responses, equity was considered between human populations, and did not mention animals nor the environment. Respondents described that pandemic prevention measures must not further widen existing human inequities, including health outcomes and economic, and should actively strive to promote equity. Similar to theme six, this is especially true between high and low income countries. This must include an effort to avoid any form of stigmatization.

*“Addressing of equity questions relating to the fact that those who are going to be most impacted by changes required for pandemic preparedness will also be some of the most disadvantaged groups globally, e.g. farmers living on marginal land”*

*“Need to be culturally and socially sensitive - Important to avoid stigmatizing”*

*“Guidelines*,* protocols*,* practices for ethical and equitable access to health service…”*.

5. Evidence-based efficiency.

Pandemic prevention measures should be based on evidence to be as efficient as possible. Without incorporating evidence into practice, limited resources could be wasted. Biomedical evidence is not the only form of evidence to consider.

*“Sensitive surveillance and response system with real time global exchange of evidences, so that the prevention/containment measures could be instituted to contain at the point source”*

*“Feasibility. Ideal interventions that are not pragmatic (whether due to resources, political aspects, or something else) will not achieve primordial prevention’s goals”*

6. Global collective effort.

All countries should be stakeholders in pandemic prevention, especially as a result of globalization, ideally through a central global agency. High income countries (HIC) need to shoulder more of the financial burden for the betterment of all, both because of the resources they have, and the disproportionate way in which they have contributed to global pandemic risk.

*“…no infectious disease risk is restricted to a certain region given globalization”*

*“Prevention strategies must involve global stakeholders*,* giving them voice and seats at the table. At present*,* international organizations like the WHO are best placed to implement such a global-scale engagement through pre-existing representative structures*.”

*“There’s a trend that HICs have historically benefitted from destroying the Earth, but LMIC bear the brunt and are chastised if they want to get their own in present-day via the same means as HICs in the past. However we move forward, HICs have to do more, not as a matter of charity or leadership, but because we’ve gotten the earth into the mess in the first place where we need to think about think long and hard about zoonotic transfers…”*

7. Intrinsic value of non-human animals and the environment:

There was agreement that the innate value of animals and the environment must be considered in pandemic prevention; However, there was variation amongst participants about the weight of their innate value as compared to the value of humanity, ranging from “some” to equal weighting as humans.

*“I would say at the very minimum the environment should be maintained for its own sake to some degree”*

*“Prevention policies should not only aim to benefit the health of human populations but they should also be in the interests of non-human animal populations that have historically shouldered the greatest burdens of human health interventions. Culling/killing healthy animal populations to mitigate risks of disease transmission to humans is not in their interest and is ethically unjustifiable”*

8. Autonomy of individuals and groups.

All levels of autonomy must be considered in pandemic prevention, from individual to nation level. When elaborated on, autonomy was in respect to human autonomy. There was no direct mention of animal or environmental autonomy.

*“Balance maximize[ing] common good while minimizing restrictions on individual freedoms”*

*“Individual freedom of choice of how to live a good life”*

*“Some interventions may be effective, but unjustifiably violate individuals’ rights or other delimiting factors”*

### Second-round

After the second-round, there was participant agreement on five of the first-round’s ethical considerations of primordial pandemic prevention policy. These included: Promoting equity, global collective effort, distributive justice, evidence based efficiency and the interconnectedness of humans, animals and the environment. There were three ethical considerations where partial agreement was achieved: Communities as stakeholders, the intrinsic value of non-human, animals and the environments, and the autonomy of individuals and groups. There were no considerations where some degree of agreement was not achieved.

As there were 25 participants in the second-round, the highest possible score an ethical consideration could achieve was 175 (25 × 7). Promoting equity received the highest total score at 148, as well as the most ratings of 7, twelve total. Global collective effort had the second highest score at 146, and second highest total ratings of 7, eleven total, but had the highest number of combined 5 to 7 ratings. Of the ethical considerations where agreement was achieved, the interconnectedness of humans, animals and the environment had the lowest total score at 140, and was in the middle of the other considerations for total number of ratings of 7, ten total. Of all the considerations, the autonomy of individuals and groups had the lowest score of 106, the lowest number of ratings of 7, two total, and the lowest number of combined 5 to 7 ratings. The complete results of the second-round are presented in Table 2.

**Table 2:** Results of second-round

	Promoting equity	Global collective effort	Distribu-tive justice	Evidence-based efficiency	Interconnectedness of humans, animals and the environment	Communities as stakeholders	Intrinsic value of non-human animals and the environment	Autonomy of individuals and groups
Total Score	148	146	143	140	140	125	123	106
# of 7	12	11	9	7	10	4	7	2
# of 6	7	6	6	9	5	5	5	2
# of 5	2	6	6	5	4	7	3	6
# of 4	2	0	3	2	2	7	3	7
# of 3	1	0	0	1	4	0	3	6
# of 2	0	1	1	0	0	2	4	2
# of 1	1	1	0	1	0	0	0	0
# of 5–7 (%)	21 (84)	23 (92)	21 (84)	21 (84)	19 (76)	16 (64)	15 (60)	10 (40)
# of 1–3 (%)	2 (8)	2 (8)	1 (4)	2 (8)	4 (16)	2 (8)	7 (28)	8 (32)

There were few open-ended comments provided in the second-round. It was noted that the “well-being (of humans, non-human animals and/or the environment) is oddly missing, and should be crucial to any response” and that “tackling the social determinants of health” could be explicitly listed as a consideration on its own, rather than included in the considerations already listed. Additionally, one responded commented that “these values immediately create conflict” with each other, and another that though “all considerations [are] important – some [are] more practical/achievable than others”.

## Discussion

This is the first survey-based study known to the authors that has conducted primary research on the ethical considerations of primordial pandemic prevention. There were five ethical considerations where consensus was achieved: Promoting equity, global collective effort, distributive justice, evidence based efficiency and the interconnectedness of humans, animals and the environment. As the purpose of our study was to determine which ethical principles should be incorporated into primary pandemic prevention, any ethical principle where agreement was achieved should be considered and prioritized in pandemic prevention policy. Given how close the total scores of the top five ranked ethical considerations were (ranging from 148 to 140 out of 175), their rank order may not be meaningful.

Interestingly, despite the One Health lens of our research question, only two of the eight themes incorporated animal and environmental perspectives, specifically the interconnectedness between animals, humans and the environment and the intrinsic value of non-human animals and the environment. The fields of public health ethics, global health ethics, and pandemic prevention, three of the four fields of participant expertise, are generally more human health focused, which could explain these results. In the second round, 72% of respondents identified as having expertise in public health ethics, which could explain why consensus was only partially achieved for the intrinsic value of non-human animals and the environment. We wonder, if with further prompting, ethical principles such as justice, autonomy and equity could have been expanded to animal and environmental considerations in pandemic prevention. Both distributive and eco justice incorporating animal and environmental perspectives have already been brought forth in the pandemic prevention literature [15, 16]. The considerations of animal autonomy, as part of a proposal for a “Belmont Report for Animals” has also been described [31].

Of the more anthropocentrically centered ethical principles uncovered, all but communities as stakeholders and the autonomy of individuals and groups achieved consensus. This was an unsurprising result for the autonomy of individuals and groups as this consideration was the least noted in the first-round. However, community as stakeholders was in the top three themes noted in the first round. This theme was captured by bringing together the concepts of honesty, transparency, and stakeholder involvement, as it was noted by respondents that it is would be unethical to conduct pandemic prevention work without informing those who may be most impacted by a prevention method. The grouping of transparency, honesty and communities as stakeholders has been previously described in the literature [17].

There were no ethical considerations for which consensus was not achieved, leaving room for further deliberation on the three considerations with partial consensus. It is possible that if additional rounds were conducted or if the study had a larger sample size, a clearer direction for communities as stakeholders, the intrinsic value of non-humans animals and the environment, and the autonomy of individuals and groups would have been determined.

We do note the lack of beneficence and non-maleficence in the results, despite these concepts being foundational to bioethics in the Beauchamp and Childress principlism model [18]. Almost all the responses that mentioned beneficence, non-maleficence, or related terms in the first-round used them in the context of distributing benefits and harms fairly, which was interpreted as distributive justice. Further research is required to determine with greater specificity the theory or theories of justice that are most relevant to pandemic prevention.

As noted by one of the participants in open-ended section of the second round, some of the ethical principles have the potential to conflict with each other. For example, as is common in public health ethical dilemmas, it can be challenging to protect autonomy while also promoting equity and distributive justice. As is the case in general ethical principlism, we suggest each principle be considered prima-facie, that no principle be considered primary, rather examined through reflective equilibrium, and that principles should be contextualized and applied through specification [18, 32].

Strengths of this study include the wide distribution of participant representation. All continents had representation in either the first and/or second-rounds. Additionally, in both rounds, there were participants from all the aimed fields of study. Future research could implement stratified sampling to ensure balanced geographic and disciplinary representation. The experience of the participants is an added strength, with at least 75% having ten or more years of experience in both rounds. Furthermore, the robust methodology of the Delphi method, based on a previous study’s protocol, added to the rigor of this study [30]. The use of the Delphi method allowed participation from across the globe and across all relevant fields of expertise, essential to a study on pandemic prevention using a One Health lens, an innately global and interdisciplinary field.

The small sample size is one of the study’s limitations, though as mentioned, the ideal number of participants in a Delphi study is not clearly defined, and we did reach our target sample number [23]. Reminder emails were sent to potential participants, as per best practice [25]. Given the relevance of the participant’s fields of work to pandemics, it is likely that many participants were occupied by the COVID-19 pandemic, which was in a global emergency state during the data collection phase of this study.

The snowball technique was used to extend of the reach of the study and increase the number of participants, however, the use of the snowball technique introduced bias into the study as it partially compromised the blindness of the participants to each other. Though participants did not know who ultimately completed the study, they would have been aware of the potential participation of anyone they referred the study to, which could introduce group conformity bias, as well as authority bias if the referrer was in a position of authority to the referee. Additionally, the snowball technique may have introduced sampling bias, skewing participation towards those who referred the study to their peers leading to the oversampling of certain respondents. In successive survey rounds, each of these biases would have been compounded. It unknown how this may have impacted consensus, as the researchers do not know who, if anyone, referred the study.

As this study aimed for global participant representation, it would have been enhanced if the questions had been translated into multiple languages. Unfortunately, our team did not have the capacity to do so, but this could be considered as a future direction. Additionally, the use of continents, rather than sub-regional divisions, may have resulted in an overgeneralization of participant representation. In future, larger studies, finer-tuned geographic categories should be used. With enough power, subgroup analysis of themes by geographic category and/or discipline could also be conducted. Future studies should include community representatives as study participants. Our research team did not have access to community representatives, but as emphasized in our study, community members are essential stakeholders in pandemic prevention work.

Practical application of our study results include applying the ethical considerations identified in current and future pandemic prevention planning. At the time of writing, the WHO completed draft zero of a pandemic prevention agreement [8]. Based on the potential widespread implications of such an agreement, it is imperative that ethical considerations be integrated into any proposed agreement. If ethics, especially with a One Health lens, are not considered, we risk missing the opportunity to support interdisciplinary collaboration to maximize benefit to humans, other animals and non-sentients, and widen existing disparities of all kinds, human to human and human to animal/environment [15]. From our findings, ensuring a just distribution of benefits and harms to global populations, promoting equity, especially in LMIC, ensuring that policy is evidence based and done collaboratively across global agencies, and the connection between human, animal and environmental health should specifically be considered.

## Conclusions

This Delphi study aimed to determine the ethical considerations of primordial pandemic prevention from a One Health perspective. With input from a globally representative sample, consensus was achieved for five ethical considerations: Promoting equity, global collective effort, distributive justice, evidence-based efficiency and the interconnectedness of humans, animals and the environment. Our findings will contribute to current and future pandemic prevention policy, and expand ethics research in the fields of One Health, pandemic prevention and zoonotic disease control.

## Electronic supplementary material

Below is the link to the electronic supplementary material.

Supplementary Material 1

## Data Availability

The raw datasets may be found on https://datadryad.org/stash/share/OQKpuscuXxdFo8ALUCkbaJ1ip4c_eCIlEZldaT9Z36A. Any additional information is available upon request.

## Abbreviations

AFMC: Association of Faculties of Medicine of Canada
COVID-19: Coronavirus disease 2019
HIC: High income countries
ILRI: International Livestock Research Institute
IPBES: Intergovernmental Science-Policy Platform on Biodiversity and Ecosystem Services
LMIC: Low and middle income countries
UNEP: United Nations Environment Programme
WHO: World Health Organization

## References

[1] Daszak P, Amuasi J, das Neves CG, Hayman D, Kuiken T, Roche B, Zambrana-Torrelio C, Buss P, Dundarova H, Feferholtz Y, Földvári G, Igbinosa E, Junglen S, Liu Q, Suzan G, Uhart M, Wannous C, Woolaston K, Mosig Reidl P, O’Brien K, Pascual U, Stoett P, Li H, Ngo. Workshop report on Biodiversity and Pandemics of the Intergovernmental Platform on Biodiversity and Ecosystem Services (IPBES). 2020.

[2] World Health Organization. WHOCoronavirus(COVID-19)Dashboard. [updated 2024; cited 2025 Jan23]. Available from: https://covid19.who.int/

[3] Organisation for Economic Co-operation and Development. Preparing for the next pandemic: What development assistance committee members should know 2022.

[4] World Health Organization. Pandemic prevention, preparedness and response accord. [2023; cited 2023 Oct 9]. Available from: https://www.who.int/news-room/questions-and-answers/item/pandemic-prevention--preparedness-and-response-accord

[5] AFMC Primer on Population Health. Glossary. 2014. Available at: https://phprimer.afmc.ca/en/glossary. Accessed March 29, 2024.

[6] AFMC Primer on Population Health. Chapter 4 Basic concepts in prevention and health promotion. 2018. Available at: https://phprimer.afmc.ca/en/part-i/chapter-4/. Accessed November 2, 2021.

[7] WHO Director-General’s opening remarks at first meeting of the Intergovernmental. Negotiating body to draft and negotiate a WHO convention, agreement or other international instrument on pandemic prevention, preparedness and response– 24 February 2022. Premium Official News 2022.

[8] World Health Organization. Zero draft of the WHO CA + for the consideration of the Intergovernmental Negotiating Body at its fourth meeting. 2023.

[9] Priyadarsini SL, Suresh M, Huisingh D. What can we learn from previous pandemics to reduce the frequency of emerging infectious diseases like COVID-19? Global Transitions. 2020;2:202–20. 32984800 10.1016/j.glt.2020.09.003 PMC7508551

[10] Philavong C, Pruvot M, Reinharz D, Mayxay M, Khammavong K, Milavong P, et al. Perception of health risks in Lao market vendors. Zoonoses Public Health. 2020;67(7):796. 32812389 10.1111/zph.12759 PMC7461205

[11] Karesh WB, Noble E. The Bushmeat Trade: increased opportunities for transmission of zoonotic. Mt Sinai J Med. 2009;76:429–34. 19787649 10.1002/msj.20139

[12] United Nations Environment Programme and International Livestock Research Institute. Preventing the Next Pandemic: Zoonotic diseases and how to break the chain of transmission. 2020.

[13] Herten v, Joost, Bovenkerk B, Verweij M. One health as a moral dilemma: towards a socially responsible zoonotic disease control. Zoonoses Public Health. 2019;66(1):26–34. 30390380 10.1111/zph.12536 PMC7379490

[14] World Health Organization. One Health [2023; cited 2023 Oct 9]. Available from: https://www.who.int/health-topics/one-health#tab=tab_1

[15] Diller E, Williamson L. Supporting one health for pandemic prevention: the need for ethical innovation. J Bioethical Inq. 2023;20(3):345–352. 10.1007/s11673-023-10264-5 PMC10235835 37266851

[16] Lysaght T, Capps B, Bailey M, Bickford D, Coker R, Lederman Z, et al. Justice is the missing link in one health: results of a mixed methods study in an urban city state. PLoS ONE. 2017;12(1):e0170967. 28129409 10.1371/journal.pone.0170967 PMC5271361

[17] Joost Hv, Buikstra S, Bovenkerk B, Stassen E. Ethical decision-making in zoonotic disease control: how do one health strategies function in the Netherlands? J Agric Environ Ethics. 2020;33(2):239–59.

[18] Beauchamp TL, Childress JF. Principles of biomedical ethics. Eighth edition. ed. New York, NY: Oxford University Press; 2019.

[19] The Lancet Global Health. WHO’s pandemic treaty: promises of equity should be kept. Lancet Global Health. 2023;11(4). 10.1016/S2214-109X(23)00121-3 36870357

[20] Brown BB. Delphi process: a methodology used for the elicitation of opinions of experts. 1968.

[21] Dalkey N, Helmer O. An experimental application of the DELPHI method to the use of experts. Manage Sci. 1963;9(3):458–67.

[22] Rowe G, Wright G. The Delphi technique as a forecasting tool: issues and analysis. Int J Forecast. 1999;15(4):353–75.

[23] Skulmoski GJ, Hartman FT, Krahn J. The Delphi method for graduate research. J Inform Technol Educ. 2007;6:1–21.

[24] Brady SR. Utilizing and adapting the Delphi method for use in qualitative research. Int J Qualitative Methods. 2015;14(5):160940691562138.

[25] Burns KEA, Duffett M, Kho ME, Meade MO, Adhikari NKJ, Sinuff T et al. A guide for the design and conduct of self-administered surveys of clinicians. Can Med Association J (CMAJ). 2008;179(3):245–52. 10.1503/cmaj.080372 PMC2474876 18663204

[26] Harris PA, Taylor R, Thielke R, Payne J, Gonzalez N, Conde JG. Research electronic data capture (REDCap)—a metadata-driven methodology and workflow process for providing translational research informatics support. J Biomed Inform. 2009;42(2):377–81. 18929686 10.1016/j.jbi.2008.08.010 PMC2700030

[27] Harris PA, Taylor R, Minor BL, Elliott V, Fernandez M, O’Neal L, et al. The REDCap consortium: building an international community of software platform partners. J Biomed Inform. 2019;95:103208. 31078660 10.1016/j.jbi.2019.103208 PMC7254481

[28] Braun V, Clarke V. Using thematic analysis in psychology. Qualitative Res Psychol. 2006;3(2):77–101.

[29] Kiger ME, Varpio L. Thematic analysis of qualitative data: AMEE Guide 131. Med Teacher. 2020;42(8):846–54. 10.1080/0142159X.2020.1755030 32356468

[30] Soriano JB, Murthy S, Marshall JC, Relan P, Diaz JV. A clinical case definition of post-COVID-19 condition by a Delphi consensus. Lancet Infect Dis. 2022;22(4):e102–7. 34951953 10.1016/S1473-3099(21)00703-9 PMC8691845

[31] Ferdowsian H, Johnson LSM, Johnson J, Fenton A, Shriver A, Gluck J. A Belmont report for animals? Camb Q Healthc Ethics. 2020;29(1):19–37. 31581963 10.1017/S0963180119000732

[32] Keeling M, Bellefleur O. Principlism and frameworks in public health ethics. Natl Collaborating Centre Healthy Public Policy. 2016;2740.

