# Supplementary material for: The ethical considerations of primordial pandemic prevention from a one health perspective
Source: Philos Ethics Humanit Med. 2025 Mar 12;20:4. doi: 10.1186/s13010-025-00166-2 (PMC11900536; doi:10.1186/s13010-025-00166-2)
Supplement: Supplementary file 1 — Supplementary Material 1 [file 13010_2025_166_MOESM1_ESM.docx]

**First Round Questions**

Demographic Questions:

Field of Expertise (Circle all that apply)

- One Health ethics
- Public health ethics
- Global health ethics
- Pandemic prevention
- Other

Geographic Region of Practice (Circle all that apply)

- Africa
- Antarctica
- Asia
- Europe
- North America
- Oceania
- South America

Years of Experience

- < 5 years
- 5-10 years
- 10-20 years
- >20 years

Study Questions:

1)  What are 3-5 **general ethical** considerations (if any) that should be included in primordial pandemic prevention policy?

2)  Which are 3-5 **global health ethical** considerations (if any) that should be included in primordial pandemic prevention policy? Note: May be the same as above

3) What are 3-5 **animal and/or environmental ethical** considerations (if any) that should be included in primordial pandemic prevention policy? Note: May be the same as above

**Second Round Questions**

Demographic Questions:

Field of Expertise (Circle all that apply)

- One Health ethics
- Public health ethics
- Global health ethics
- Pandemic prevention
- Other

Geographic Region of Practice (Circle all that apply)

- Africa
- Antarctica
- Asia
- Europe
- North America
- Oceania
- South America

Years of Experience

- < 5 years
- 5-10 years
- 10-20 years
- >20 years

Study Questions:

Based on the responses from the Round 1 Survey, the following eight themes were identified as ethical considerations of primordial prevention* of the next global pandemic from a One Health perspective, in no particular order:

1. Intrinsic **value** of non-human animals and the environment: There is agreement that the innate value of animals and the environment must be considered in pandemic prevention. There is uncertainty about the weight of their innate value as compared to the value of humanity.
2. **Interconnectedness** of humans, animals and the environment: Human, animal and environmental health overlap, which must be considered both in each of their independent innate value as well as in pandemic prevention for the sake of human health.
3. **Communities** as stakeholders: Community members must be included as stakeholders in decision making. Methods of doing so include active engagement with the community, public education, maintaining public trust and being publically transparent in decision-making.
4. **Global** **collective** effort: All countries should be stakeholders in pandemic prevention. Ideally through a central global agency. HIC need to shoulder more of the financial burden for the betterment of all.
5. Promoting **equity**: Pandemic prevention measures must not further widen existing inequities, and should actively strive to promote equity. This is especially true between H and L IC, and must include an effort to avoid stigmatization.
6. **Distributive Justice**: **Fair** distribution of **benefits** and **burdens:** The benefits and burdens of pandemic prevention must be distributed in a manner that is fair based on the ethical considerations in this list. This could include between nations, groups of people, generations, species etc.
7. **Evidence-based efficiency**: Pandemic prevention measures should be based on evidence to be as efficient as possible. Biomedical evidence is not the only form of evidence to consider.
8. **Autonomy** of individuals and groups: All levels of autonomy must be considered in pandemic prevention, from individual to nation level.

1. Please rate the following using a 7 point Likert scale (1= least important, 7=most important). Multiple ethical considerations may receive the same numerical value if they are considered of equal importance level.

1. Intrinsic **value** of non-human animals and the environment
2. **Interconnectedness** of humans, animals and the environment
3. **Communities** as stakeholders
4. **Global** **collective** effort
5. Promoting **equity**
6. **Distributive Justice**
7. **Evidence-based efficiency**
8. **Autonomy** of individuals and groups

2. Please advise if there are any ethical considerations that are missing from this list:

3. Provide additional comments here:
